# “They are saying it’s high, but I think it’s quite low”: exploring cardiovascular disease risk communication in NHS health checks through video-stimulated recall interviews with patients – a qualitative study

**DOI:** 10.1186/s12875-024-02357-w

**Published:** 2024-04-23

**Authors:** Lisa Cowap, Victoria Riley, Sarah Grogan, Naomi J. Ellis, Diane Crone, Elizabeth Cottrell, Ruth Chambers, David Clark-Carter, Christopher J. Gidlow

**Affiliations:** 1 Staffordshire University, Stoke-on-Trent, UK; 2 Manchester Metropolitan University, Manchester, UK; 3 Cardiff Metropolitan University, Cardiff, UK; 4 Wolstanton Medical Centre, Newcastle-under-Lyme, UK

**Keywords:** Cardiovascular diseases, Risk, Preventive medicine, Primary health care, Qualitative research

## Abstract

**Background:**

NHS Health Check (NHSHC) is a national cardiovascular disease (CVD) risk identification and management programme. However, evidence suggests a limited understanding of the most used metric to communicate CVD risk with patients (10-year percentage risk). This study used novel application of video-stimulated recall interviews to understand patient perceptions and understanding of CVD risk following an NHSHC that used one of two different CVD risk calculators.

**Methods:**

Qualitative, semi-structured video-stimulated recall interviews were conducted with patients (*n* = 40) who had attended an NHSHC using either the QRISK2 10-year risk calculator (*n* = 19) or JBS3 lifetime CVD risk calculator (*n* = 21). Interviews were transcribed and analysed using reflexive thematic analysis.

**Results:**

Analysis resulted in the development of four themes: variability in understanding, relief about personal risk, perceived changeability of CVD risk, and positive impact of visual displays. The first three themes were evident across the two patient groups, regardless of risk calculator; the latter related to JBS3 only. Patients felt relieved about their CVD risk, yet there were differences in understanding between calculators. Heart age within JBS3 prompted more accessible risk appraisal, yet mixed understanding was evident for both calculators. Event-free survival age also resulted in misunderstanding. QRISK2 patients tended to question the ability for CVD risk to change, while risk manipulation through JBS3 facilitated this understanding. Displaying information visually also appeared to enhance understanding.

**Conclusions:**

Effective communication of CVD risk within NHSHC remains challenging, and lifetime risk metrics still lead to mixed levels of understanding in patients. However, visual presentation of information, alongside risk manipulation during NHSHCs can help to increase understanding and prompt risk-reducing lifestyle changes.

**Trial registration:**

ISRCTN10443908. Registered 7th February 2017.

**Supplementary Information:**

The online version contains supplementary material available at 10.1186/s12875-024-02357-w.

## Introduction

Cardiovascular disease (CVD) is a global concern and accounts for a quarter of premature deaths in the United Kingdom [UK; 1]. Given the apparent plateau in declining CVD-related deaths [2], CVD prevention is a strategic priority for the National Health Service [NHS; 3], for which the NHS Health Check (NHSHC) programme is an important part [3, 4]. This national programme is offered to all adults in England aged 40–74 years who do not have specific pre-existing circulatory conditions [5]. The NHSHC, typically delivered in primary care by a primary care nurse [practice nurse; PN, or health care assistant; HCA], should involve assessment of CVD risk, communication of that risk, and discussion around its management [5].

A national review of the NHSHC programme in 2020 highlighted some potential changes and additions over the next 10 years, such as the introduction of digital NHSHC that could be performed at home, including assessment of other common chronic conditions, such as mental health and musculoskeletal health problems, and a lowering of the minimum age for eligibility to 30 years [6]. Despite a clear intention to reduce the need for in-practice, face-to-face NHSHC, these consultations and the assessment of CVD risk will remain an important part of the programme.

In line with National Institute for Health and Care Excellence (NICE) guidance [7] and as a programme requirement [5], NHSHC practitioners assess and communicate CVD risk using QRISK (QRISK2, now superseded by QRISK3). This is presented as a 10-year percentage risk estimate of heart attack or stroke. However, the limitations of such short-term absolute risk metrics are well-documented. They include the strong influence of age that leads to under-/over-estimation of risk in the young and old, respectively [8, 9] and lack of understanding among patients and practitioners [10–15]. This might have negative implications for recall and resulting behavioural choices among patients to improve or manage their CVD risk.

The JBS3 risk calculator was developed as an alternative [8], with a focus on lifetime CVD risk and includes various interactive, visual presentations that aim to better facilitate patient and practitioner understanding of CVD risk (than QRISK). JBS3 includes lifetime risk scores, such as heart age - the estimated age of a person of the same gender, ethnicity, and risk of an annual event, but with all other CVD risk factors at ‘optimal’ levels [8]. It also includes event-free survival age, which estimates the age an individual can expect to reach without having a CVD event. Unlike QRISK, JBS3 presents CVD risk scores using various visual representations and allows manipulation of risk scores through modifying risk factors (e.g., smoking status, weight, blood pressure) to demonstrate how intervention can reduce CVD risk. Such interactive graphics can be beneficial through engaging individuals with the information, promoting understanding and retention [16, 17], yet the impact for communication of CVD risk remains unknown.

Despite the potential improvements to understanding of CVD risk that JBS3 may facilitate, perceptions and understanding from a patient perspective are yet to be qualitatively explored in comparison to ‘usual care’ (i.e., CVD risk communicated via 10-year percentage risk using QRISK2). The RIsk COmmunication in NHSHC (RICO) study was a large-scale qualitative study that used video-recording methods to explore how NHSHC patients and practitioners understood and discussed CVD when using QRISK2 and the potential for JBS3. Key findings from quantitative and qualitative assessment of consultations suggest that NHSHC using QRISK2 (usual care) tend to be quicker, with more practitioner-dominated speech and less CVD risk discussion than those using JBS3 [18, 19]. Video-stimulated recall (VSR) interviews with practitioners identified a mismatch between the expectations and reality of their understanding, competencies and training around CVD risk communication [20]. Specifically, despite apparent confidence in delivering the QRISK2 10-year risk scores, such scores were not well understood by practitioners. They were regarded primarily as a means of identifying patients as low, medium and high-risk to guide clinical decision-making around routine medical follow-up, rather than a tool to facilitate a discussion of CVD risk with patients [20]. Ultimately, we observed a lack of understanding and confidence in explaining 10-year risk among NHSHC practitioners.

This paper presents data from VSR interviews with NHSHC patients from the RICO study. As reported elsewhere, this methodology had not been previously applied to NHSHCs but is well-suited to explore topics with complexity such as CVD risk communication through patient-practitioner interactions [21, 22]. Video-stimulated recall interviews go beyond the traditional semi-structured interview in that video clips from a consultation can be used to aid recall, thus exploring participant perceptions prior to the clip being shown, and then afterwards to gain deeper insight into conversations that might not be easily recalled. This leads to more meaningful discussion of topics, going beyond general descriptions of the event [23].

## Method

### Aim

We aimed to qualitatively explore patient perceptions of CVD risk during an NHSHC using two different risk calculators; QRISK2 and JBS3.

### Design

The RICO study was a qualitative exploration of patient and practitioner understanding of CVD risk using two different risk calculators: QRISK2 and JBS3, during NHSHCs in general practice. Full details of the study design and protocols are reported elsewhere [24].

### Setting

Data collection took place between January 2018 to February 2019 in 12 general practices from across the West Midlands of England, UK. Six pairs of practices, approximately matched by deprivation, were randomly allocated to deliver NHSHCs using JBS3 to communicate CVD risk (intervention) or to continue using QRISK2 (usual practice). Two of the six practices allocated to ‘usual practice’ used Informatica, additional software with some of the features of JBS3, such as the heart age and a risk score manipulation function. These were retained and data were included in analysis to give a true representation of ‘usual care’ and are highlighted using the term QRISK2+.

### Participants

Participants (*n* = 40) were a subsample of patients who received an NHSHC through the RICO study, in which over 170 NHSHCs were video-recorded. All participants were eligible for an NHSHC according to criteria (aged 40–74 years, no pre-existing circulatory conditions, not received NHSHC in last five years, not on statins, not known to be at high risk of CVD [5]). Patient recruitment into the main study involved participant stratification by age, gender and ethnicity [24]. The VSR interview sample were stratified as far as possible by age group (40–54/55–64/65–74 years), gender (male/female), ethnicity (white British/ethnic minorities) and level of CVD risk (low/medium-high) to provide a demographic range and balance across JBS3 and QRISK2 groups.

### Procedures

Participants identified through stratification were invited to take part in a semi-structured, one-to-one VSR interview at their general practice within the four weeks following their NHSHC. All processes and materials were piloted prior to data collection through Public and Patient Involvement and Engagement (PPIE) for the RICO study [25]. Interview schedules were tailored to each group (QRISK2 or JBS3 – please see Supplementary files one and two). For each risk format (e.g., percentage risk score, heart age, event-free survival age, risk score manipulation), initial questions explored patient recall (e.g., recollection of being told CVD risk score), then video clips were shown to elicit more in-depth discussion about their experiences. For each VSR interview participant, the recording of their NHSHC was reviewed by two researchers (LC, VR) to identify relevant parts to use as excerpts for VSR interviews, which included: discussion of CVD risk (particularly the CVD risk score); lifetime risk and risk modification (for those in the JBS3 group); and practitioner recommendations for CVD risk management. Participants in the QRISK2 group were also shown JBS3 outputs to explore their perceptions of JBS3-specific features. Mean interview duration was 34:59 min (SD = 8:53; range 19:35 to 54:25).

Interviews were conducted by two Caucasian, female researchers (LC, VR), residing in the Northwest and Midlands of England, UK, respectively. Both researchers have extensive interview experience with a range of participant groups, including interviews in primary care settings. LC is a Health Psychologist registered with the Health and Care Professions Council (HCPC), a Lecturer in Health Psychology and has research experience predominantly related to children’s healthy eating and adolescent smoking behaviours. VR is a Research Associate and has research experience related to NHSHC and CVD risk communication. No other non-participants were present during data collection. All interviews were audio-recorded and transcribed verbatim.

### Analysis

Transcripts were imported into NVivo 12 software to facilitate analysis. Data were analysed using inductive, reflexive Thematic Analysis following Braun and Clarke’s guidelines [26–28], from a critical realist epistemological standpoint. Transcripts were initially read and re-read during familiarisation to gain greater understanding of the data. Line-by-line coding was completed by the first author (LC) primarily at a semantic level. The data were firstly described before moving on to code at a more latent level, summarising and then considering patterns across data. This process was cyclical in nature and constant comparisons were made between the data and generated themes [29]. Final themes were discussed with a second author (VR) who had been immersed in data collection and confirmed by a third author (SG), an expert in qualitative research methods, with more than 30 years of qualitative research experience. The resulting themes were agreed by all authors.

## Results

### Participant characteristics

The final sample was approximately evenly distributed by gender and age groups overall, although with some differences between groups (e.g., higher proportions of participants who were male, 65–74-year-olds, white British, or medium-high CVD risk in JBS3 compared with QRISK2; Table 1).

**Table 1:** Patient characteristics for video-stimulated recall interviews

		Total		JBS3		QRISK2	
		n	%	n	%	n	%
Age (years)	40–54	14	35.00	6	28.57	8	42.11
	55–64	14	35.00	7	33.33	7	36.84
	65–74	12	30.00	8	38.10	4	21.05
	Total	40		21		19	
Gender	Male	21	52.50	13	61.90	8	42.11
	Female	19	47.50	8	38.10	11	57.89
	Total	40		21		19	
Ethnicity	White British	36	90.00	18	85.71	18	94.74
	Ethnic minority	4	10.00		14.29		5.26
	Total	40		21		19	
CVD risk category	Low (< 10%)	28	70.00	13	61.90	15	78.95
	Medium-High (≥ 10%)	12	30.00	8	38.10	4	21.05
	Total	40		21		19	

A full report of the results is available elsewhere [30]. Analysis resulted in four themes: ‘variability in understanding’, ‘relief about personal risk’, ‘perceived changeability of CVD risk’ and ‘positive impact of visual displays’. The first three themes were evident across the two patient groups, regardless of risk calculator; the latter related to JBS3 only. To clarify the relevance of findings for practice as well as research, findings are presented by risk calculator, using illustrative quotations and some interviewer-patient exchanges. Quotations are labelled to show gender, age, participant number and CVD risk calculator (QRISK2 + where Informatica was used). Where dialogue is reported, ‘I’ denotes the interviewer’s contribution, and ‘P’ denotes the patient’s contribution. Given that 10-year percentage risk information is provided regardless of the risk calculator experienced, related perceptions from all participants are included.

### 10-year percentage risk calculator (QRISK2)

#### Relief about personal risk

Most patients demonstrated a lack of concern. As many patients’ QRISK2 scores were under 10%, they found it “reassuring… it was under 10… I was just relieved” (Female, 56, 3_125, QRISK2). However, perceiving 10-year CVD risk as “small” or “quite low” was not limited to those classified as low risk. Patients dismissed their CVD risk if they perceived it to be low even if, as here, the practitioner had suggested that 10-year risk was slightly raised:

You tend to look at it and then tend to be kind of… brush it away… although she said it was quite high, I think because I think it is such a low percent, it’s kind of not at the front of my mind and I am not concentrating on it. (Female, 39, 9_083, QRISK2)

It was common for patients to explain that a higher CVD risk would have caused more concern and be more likely to prompt change. For example, one patient with a 10-year risk of 15% (moderate) described how he would have been sufficiently worried to act if it had been double (i.e., very high). This highlights that greater understanding is needed for an appropriate appraisal of CVD risk, that could lead to risk-reducing behaviour: “You know, I mean if you’d said to me it was 30% …obviously I’d be concerned about it…or listened more, or look at taking steps, to… rectify it or do something about it” (Male, 58, 11_028, JBS3). Furthermore, patient accounts often illustrated a mismatch between the expectation and reality of CVD risk. Some expected their overall CVD risk would be higher than it was based on known risk factors: “I thought [10-year risk] were going to be higher, because of … my blood pressure and the cholesterol, and so I thought “oh I am in trouble here” … I really did think it was going to be high” (Female, 56, 3_125, QRISK2). This could be falsely reassuring to patients and undermine their perceived vulnerability to developing CVD, particularly with already elevated risk factors.

#### Variability in understanding

Perceived understanding of CVD risk was varied. Some patients thought they understood the information provided to them (despite apparent misunderstanding):

I: Do you understand what [practitioner] meant when she gave you that percentage score?

P: Yeah that you’ve got 6% risk of, of getting heart disease in life really yeah, out of 100 people you know 1 in 6. (Female, 57, 12_131, QRISK2+)

Others felt confused and struggled to put the percentage score in the context of a ‘good’ or ‘bad’ outcome, regardless of risk category (low, medium, high). For example, a low-risk patient described: “I think if 3% is a high risk of you know heart disease, then 3% is not good, because 3% makes you think it’s good, because 3 out of 100 is good” (Female, 51, 9_295, QRISK2). Similarly in a patient with high CVD risk: “I can’t quite understand what like 25% is, what’s, what’s good and what’s bad with 25%?” (Male, 74, 5_132, JBS3).

Despite failing to understand or even remember what practitioners told them about their 10-year risk, patients often described it positively: “obviously I didn’t recall it, but I did think that was good how that was explained and showed” (Male, 57, 4_080, JBS3). But any related benefits could be undermined by the lack of recall or understanding of CVD risk information. Providing further context to demonstrate the relevance of CVD to patients (its severity or their vulnerability to it) was suggested to improve understanding of 10-year risk and its implications:

I think with the percentage unless you have been given the range it should be in for your age and for your, you know, capabilities, then it’s kind of a mismatch of information…they are saying it’s high, but I think it’s quite low, but I don’t know what high is because I haven’t been given anything to compare it against. (Female, 39, 9_083, QRISK2)

#### Perceived changeability of CVD risk

There was varied understanding about whether 10-year risk could be changed by modifying lifestyle factors. Some patients believed that their percentage score was fixed: “the fact that my dad had a heart attack has increased my score… and she said herself on the video, you can’t do anything about that” (Male, 61, 6_044, QRISK2). Most recognised that risk could be modified; that it will increase over time: “she did say that it wouldn’t stay the same it would change, I do need to start looking after me self” (Male, 51, 10_539, QRISK2), and that it could be reduced through behaviour change: “it could come down, it could be better” (Female, 66, 2_001, QRISK2+). But patients were not always clear how: “right now so it’s 4.8% over the next 10 years, but I don’t know how you would lessen that” (Female, 61, 4_263, JBS3).

The idea of CVD risk being a prediction, or a ‘lottery’ was also evident and appeared detrimental to patient’s perceived ability to make risk-reducing changes:

To me it didn’t mean anything, because to me you know I can change my lifestyle and all that sort of thing, but at the end of the day it is a bit of a sort of like lottery really, isn’t it? (Female, 66, 2_001, QRISK2+)

This highlights an important separation in many patient’s minds between their lifestyle/behaviour and future disease risk, which might lack credibility (lottery) or be considered outside of their control (fixed). Ultimately, this makes clear the important link between clarity and understanding of CVD risk information, and patient’s perceptions (plus subsequent intentions) regarding their ability to reduce risk through lifestyle change.

### JBS3 risk calculator

#### Heart age

##### Variability in understanding

Understanding of heart age was also somewhat mixed, although the inherent comparison of heart age versus chronological age allowed some patients to quickly appraise their risk: “straight away you know whether that is good, or bad, because if your [chronological] heart age is lower than the reading [heart age estimate], then you know straight away that is not so good” (Female, 51, 9_295, QRISK2). Patient understanding of CVD risk was also increased through use of heart age: “so the model brings it up as your heart age, given the information that it has got, is 61 years and I thought ‘well that is so clear and understandable’, so I found that very helpful” (Female, 61, 4_263, JBS3). Some patients, however, failed to understand the significance of having a heart age different from their actual age: “You’ve got a heart of a 72-year-old, or 73-year-old and you’re, you’re 62. What does that actually mean?” (Male, 62, 4_143, JBS3). In such cases, providing more context and explanation could have helped: “it needs more context I think… on reflection as I walked away I don’t, didn’t really ask what that meant, it’s just stuck in me that it’s like hmm… it’s not younger than me which, I, imagined it should be” (Male, 40, 12_064, QRISK2+).

Nonetheless, heart age appeared to be the most memorable risk metric provided, and was often described as more impactful:

The thing that registered with me and that sort of really grabbed my attention… all I could see was that 65 on that screen…that was wallop…that heart age and I think perhaps I missed some of the, shall we say, the finer detail because I was focussed on that…I could see that 65 and I was thinking bloody hell I ain’t 65 (Male, 59, 7_105, JBS3).

Furthermore, knowledge of their heart age did appear to prompt change within some participants, more so than other metrics: “it is that swift kick to say ‘get out there and do something’’’ (Male, 64, 2_084, QRISK2+).

##### Relief about personal risk

Like 10-year risk, there was evidence of patients being reassured or “quite pleased” (Female, 61, 4_263, JBS3) by a heart age that was similar to their actual age. However, as the meaning and relevance of heart age were perhaps better understood, more patients were surprised by a high heart age (than by elevated 10-year risk):

That is a bit of a surprise really for that, because… I still feel quite energetic and still play you know the sports I do, I am never tired, or feeling like I can’t go on… I do the complete opposite. (Male, 57, 4_311, JBS3)

This suggests that ‘relief’ among patients was less strong for heart age, and therefore, the potential to promote risk-reducing behaviour change may be higher. Of all the methods of presenting CVD risk information, conversations around heart age were most common, and rarely in a negative context. Some patients did feel “a bit shocked” (Female, 68, 12_189, QRISK2+) after receiving this information, but still the positive impact was evident.

#### Event-free survival age

##### Variability in understanding

JBS3 event-free survival age caused most confusion. Instead of interpreting this as an estimate of the age they could expect to live free from CVD events, a small number of patients interpreted this as predicted age of survival. This caused observable concern in some patients given this lifetime risk estimate during the NHSHC:

I And it kind of stuck in your mind?

P Oh blooming heck it did… I was walking across here asking myself what did she mean there, is that it? … And I thought to myself I’ve got to get some living done in 7 years then (Male, 74, 5_132, JBS3).

Rather than fostering understanding and subsequent behaviour change, this misinterpretation seemed to have a negative effect on patient mental wellbeing in some cases as illustrated from the quote. Several patients dismissed event-free survival age on the basis that it was an estimate, but also because of their misunderstanding: “I’d take that [event-free survival age] as a pinch of salt … You can’t predict that… You know, that’s pretty ridiculous … to predict how long I’m gonna, live really… science fiction ain’t it?” (Male, 48, 9_087, QRISK2). Ultimately, patient’s discussion of event-free survival age demonstrated that they either misinterpreted the information and were alarmed, or did not understand and, therefore, did not believe it.

#### Risk score manipulation and visual displays

##### Positive impact of visual displays

The visual presentation of risk information in JBS3 was thought to overcome barriers around verbal communication as: “it was good that you could see the screen and … how she worked it out as well rather than somebody just telling you” (Female, 54, 7_044, JBS3). This strengthened the message and aided recall:

… because it was on the screen, I think that is such an aid to memory… Because in any situation that is new to you, if there’s a lot of things going on and you are not sure what’s going on, you don’t hear… But if you see it, it is actually much, much clearer to you. (Female, 61, 4_263, JBS3)

This highlights the benefit of using visual displays of CVD risk to address the common difficulty of patients feeling unable to absorb (and therefore, retain and recall) the volume of information provided within an NHSHC: “when you are in somewhere like that you can’t take on too much either can you really? Because it all becomes a bit muddled together” (Female, 612, 4_263, JBS3), particularly the numerical information: “Didn’t really sort of take in… what the numbers were” (Female, 56, 3_125, QRISK2).

##### Perceived changeability of CVD risk

Overall, the ability to manipulate CVD risk was perceived positively by patients:

Yes, I think it helps, rather than somebody talking to you… You can see it and then by altering it, you know and saying, “if we put this information in you can see how … if you were much heavier say for example, or if you smoke, or if you do these sorts of things”, so I found that really helpful. (Female, 61, 4_263, JBS3)

There was also a suggestion that patients may take more notice of the information due to this change in presentation, which influenced intentions towards risk-reducing behaviour:

I certainly got the gist of what [practitioner] was saying and it’s quite graphic seeing it there on screen erm, you know heart age 65 and I’m, I’m not quite 60 so you’re thinking, “yeah I ought to do something about that” and yeah the intention is there. (Male, 59, 7_105, JBS3)

Again, there were implications for motivating risk-reducing behaviour, with some patients having already implemented recommended changes based on their risk manipulation in JBS3 (up to 4 weeks post-NHSHC):

Yes, she did yeah bring the percentages down and all this… I took on board… I came out thinking, “well yes my lifestyle needs to change…” I have made the effort and through that, through this [NHSHC] you know so it… the benefits are there. It’s definitely done something for me’. (Male, 65, 4_394, JBS3)

This suggests that the presentation of CVD risk in this way can enhance understanding and promote required behaviour change, perhaps due to patients visually seeing how CVD risk can be modified through factors under their control.

## Discussion

### Summary

This is the first study, to our knowledge, which explored and reports on patient perspectives of CVD risk communication using QRISK2 and JBS3 in primary care, through use of VSR interviews. Although research has previously explored patient perceptions of 10-year percentage risk and heart age, the present study strengthens this knowledge, through use of novel methodology to facilitate recall and reflection of events during the NHSHC. Furthermore, patient perceptions of a novel tool to deliver CVD risk information (via JBS3), where knowledge is limited, have also been explored. This is particularly important to understand the most impactful metrics for lifetime risk, and where more work is needed to enhance communication.

In response to the study aim, four main themes were developed, demonstrating patient perspectives: ‘variability in understanding’, ‘relief about personal risk’, ‘perceived changeability of CVD risk’ and ‘positive impact of visual displays’. The first three themes were evident among patients who received an NHSHC using either risk calculator; the latter related to JBS3 only.

### 10-year percentage risk calculator (QRISK2)

Generally, findings related to 10-year percentage risk reflected the wider literature [e.g., 14, 15]. There was often a lack of concern about CVD risk, but particularly when the percentage was misinterpreted as ‘low’ risk. This supports evidence that it is uncommon for individuals to perceive themselves at high risk of developing CVD [31]. However, such perceptions often resulted in dismissal of the score by patients, even if this was high in the context of their individual elevated risk factors. However, this is unsurprising given previous findings that the NHSHC does not adequately convey the importance of the risk score [32]. It seems probable that 10-year percentage risk does not foster the appropriate level of understanding and perceived vulnerability to CVD risk, which may be required to facilitate risk-reducing behaviour change. It was common for patients to suggest that they would be more concerned if a higher percentage risk were provided. Therefore, more appropriate appraisal of this metric is required. Overall, patients appeared to misinterpret the meaning of the risk score in their appraisals, as reported elsewhere [32–35].

Understanding of 10-year percentage risk varied considerably across patients, again reflecting previous literature [36]. Some patients demonstrated understanding, whereas others felt confused and struggled to contextualise into a ‘good’ or ‘bad’ outcome. Misinterpreting the meaning of the risk score [32–35] or finding this information confusing [31, 34, 37, 38] is common in the literature. It also accords with evidence that practitioners responsible for communicating CVD risk lack understanding of this metric, with suggestions that more training is required to improve this important part of NHSHCs [20]. There was also variation in patient perceptions around the nature of CVD risk, given some considered it to be more fixed and likened it to a lottery, whereas others did recognise the ability to modify it.

### JBS3 risk calculator

Patient understanding of heart age was also mixed, although this metric did appear to be more memorable and was rarely discussed negatively. Inherent comparisons between heart age and chronological age were common and seemed to facilitate rapid appraisal of risk and increase understanding. This is reflective of the literature which suggests that heart age has the potential to promote effective CVD risk communication given the metric is easier to understand and recall by patients [39, 40], and is generally preferable to communication using absolute risk or ‘usual care’ in randomised controlled trials [41]. Yet, the quality of such trials has been questioned and additional research suggests that heart age may not be a motivating risk metric when compared to 10-year percentage risk [42]. Furthermore, like 10-year percentage risk, additional context around this metric would have further enhanced patient understanding.

Some patients reported surprise at being told their heart age, which is promising given the ability of 10-year percentage risk to provide false reassurance, particularly to younger patients. If patients are expressing surprise, it could have positive implications for engagement in required risk-reducing behaviour change, more so than in patients who feel generally happy with their CVD risk assessment. This is perhaps why previous research has found heart age to encourage patients to make adaptive lifestyle changes [41–45]. Yet communication of heart age did promote confusion and relief in some patients, like 10-year percentage risk, which does suggest it is not flaw-free. The potential for patients to experience a negative emotional response to being given their heart age is apparent, and reflective of the literature [43], yet the present study did not find evidence of inflated risk perceptions in relation to heart age as other research has [43].

A strength of JBS3 was the visual representation of CVD risk information, including the ability to visually manipulate the information based on hypothetical changes to lifestyle risk factors (e.g., show change in heart age associated with smoking cessation). Both aspects were positively perceived, particularly the visual display of information, which seemed to increase accessibility and promote understanding. This, alongside risk manipulation, was suggested to have a positive impact for motivating risk-reducing behaviour. This is perhaps not surprising given the evidence supporting benefits of presenting risk information visually [16, 17], but this was the first time it has been shown in the NHSHC setting.

An apparent limitation of JBS3 related to communication of event-free survival age. While this is intended to enhance understanding, particularly in younger patients where a 10-year percentage risk may be falsely reassuring, this concept often created confusion and concern. It was the metric least recalled by patients. Some dismissed the event-free survival age as lacking credibility (more so than other metrics), while others misunderstood and, consequently, did not believe it. Of most concern were the patients who confused event-free survival age estimates with their predicted age of death. This is the first study to explore the use of event-free survival age in an NHSHC setting. It is clear, however, that careful consideration of the most effective ways of communicating this information is required (including appropriate practitioner training), to ensure it is fully understandable, relatable and does not result in unintended consequences that could be detrimental to patient mental wellbeing.

### Strengths & limitations

This study has several strengths. Use of VSR offered a methodological advantage over more traditional methods of qualitative data collection, by encouraging patients to identify matters of importance to them and the ability to provoke a more emotive response to information discussed during a consultation [46]. Patient perceptions of CVD risk communication were also not restricted or reliant on retrospective recall of the event [46], which may be influenced by recall bias. However, there is evidence that VSR has the potential to elicit social desirability bias in some individuals [47] and can lead to alterations in practitioner behaviour [48]. Yet, this was not evident in this study. In addition, stratified sampling allowed for a more diverse participant sample in terms of age, gender, ethnicity, and level of CVD risk.

Limitations are acknowledged. First, while stratified sampling did diversify recruitment, practice population demographics and participant availability led to some differences between groups; the JBS3 group contained more males and 65–74-year-olds, patients of White British ethnicity and medium-high risk of CVD. There were also a larger number of patients with a low level of CVD risk, meaning that patients with medium-high level of CVD risk were under-represented. Finally, all patients were recruited from General Practices in the West Midlands and therefore the transferability of the findings to other geographical areas of the UK may be limited.

### Implications for practice

Overall, reliance on 10-year percentages to communicate CVD risk in NHSHCs is problematic as many patients do not understand it sufficiently to appraise their personal risk. This will ultimately not promote risk-reducing behaviour change where needed. Some discord between the tool purpose and how it is used in NHSHC is perhaps not surprising. Ten-year percentage risk scores were developed to guide medication initiation and not to aid patient understanding [39]. The video-recorded consultations confirmed that these tools are generally not used to initiate a discussion around use of statins [19]; if appropriate, this would form part of the follow-up with referral back to the GP [30]. Regardless of their 10-year percentage risk, the focus of the CVD risk discussion in NHSHC was to aid patient understanding and to encourage lifestyle change. As NICE guidance still recommends use of QRISK, it seems likely percentage 10-year risk will continue to be used in NHSHCs. If so, training is required to enhance practitioner competence and confidence in communicating the information to patients and promote understanding. The remit of NHSHC might expand to include other chronic conditions [6], but CVD risk will remain integral.

The national review of NHSHC [6] recognised the limitations of 10-year percentage risk as highlighted in this paper and acknowledges the need to improve communication of CVD risk within the NHSHC. Provision of heart age, visual representation of risk, and visual manipulation of risk factors offer other avenues of CVD risk communication, which patients may find more accessible, easier to understand and as a result, may promote behaviour change. These metrics could be used alongside 10-year percentage risk, or perhaps in place of them. Event-free survival age metrics also show theoretical promise, but more empirical work is required to understand how these metrics can be effectively communicated to maximise understanding and impact, particularly due to the lack of available literature. Further exploration of the potential impact of visualisations will also be important, given the lack of research specific to NHSHCs.

Ultimately, the literature [20] and the outcomes of this paper illustrate the importance and role of practitioner training in delivering effective CVD risk communication within NHSHCs. Regardless of the recommended approach, whether this remains as communication of 10-year percentage risk, or the introduction of lifetime risk metrics, practitioners need to be given the flexibility to personalise CVD risk discussion to meet the needs of the patient and be effectively trained in using these tools and given the appropriate time to use them properly within NHSHCs, to enhance communication. Purely providing the tool without training and sufficient time is ineffective and may perpetuate misunderstanding for both practitioners and patients.

## Conclusions

This is the first qualitative exploration of patient perspectives comparing CVD risk communication using QRISK2 and JBS3 in NHSHCs, using a novel VSR methodology. The findings overall reflect those from previous research that highlights the limitations of QRISK2. Heart age was viewed more favourably, facilitating a quick appraisal of risk and increased motivation regarding behaviour change, but the potential for confusion was evident, and event-free survival age created too much confusion and concern to be recommended at this stage. However, use of visual displays and visual manipulation of risk in JBS3 appeared to overcome communication barriers. These findings are consistent with those reflected in the national review of NHSHC [6], where improvement of risk assessment and communication is recommended. It is hoped that outcomes of the current research will be beneficial in facilitating such improvements.

### Electronic supplementary material

Below is the link to the electronic supplementary material.

Supplementary Material 1

Supplementary Material 2

## Data Availability

All data generated and analyzed during the current study are not publicly available due to the confidential nature of participant transcript data, but are available from the corresponding author on reasonable request.
