# Supplementary material for: “They are saying it’s high, but I think it’s quite low”: exploring cardiovascular disease risk communication in NHS health checks through video-stimulated recall interviews with patients – a qualitative study
Source: BMC Prim Care. 2024 Apr 23;25:126. doi: 10.1186/s12875-024-02357-w (PMC11036616; doi:10.1186/s12875-024-02357-w)
Supplement: Supplementary file 2 — Supplementary Material 2 [file 12875_2024_2357_MOESM2_ESM.docx]

Supplementary file 1: RICO VSR Interview Schedule – Patient – QRISK2

Introduction

“Hi, my name is [name]. Firstly, I would just like to thank you again for agreeing to participate in this interview. The interview will ask you a series of questions concerning your views and experiences of the NHS Health Check that you recently attended at your GP practice. You will remember that the appointment was video recorded, and we would like to show you some clips from the recording to ask you for your opinion on what happened during your consultation. We just want to understand from your perspective how the session went. There are no right or wrong answers. The interview is anonymous and so all names will be replaced with a fake name. Therefore you will not be identified in any written reports, though we will use quotations from the interviews in our reports, these will not be linked to any one person. Do you have any questions before we begin?”

- “Was it about XX weeks ago that you went for your Health Check?”
- “Was it a morning or afternoon appointment?”

1. Experiences and perceptions of the Health Check

- “Had you heard of NHS Health Checks before attending the appointment?”
  - *“What did you know before your Health Check?”*
  - *“What did you think they were about?”*
  - *“Having attended one, what in your opinion is the purpose of the programme?*
  - *“Has your understanding of the Health Check changed after attending?”*
- “Can you tell me about what happened during your Health Check?”
  - *“Why did you attend?”*
  - *“We would like to know of any good or bad things about the Health Check…”*
    - *“Were there any good things that came out of attending?”*
    - *“Were there any bad things about attending?”*
  - *“Was anything in the Health Check helpful or unhelpful?”*
    - *“What?”*
    - *“Why?”*
  - *“Was there anything that could have been said to make these more helpful/positive?” [only ask if bad or unhelpful things are previously mentioned]*
  - *“Was there anything you learnt at your Health Check?”*
    - *“Did you find anything confusing?”*

1. Risk score-related questions

- “Do you remember what you were told about your level of cardiovascular disease risk?”
- “You were probably given a percentage risk score, do you remember what that was?”
  - *“If yes, can you remember how you felt about it?”*
  - *“If no, don’t worry if you can’t remember, I’m about to show you a clip of what was talked about”.*

**[Show video excerpt – risk score]**

- “After seeing the clip, how are you feeling now about hearing/seeing the score?”
  - “Has your feelings changed now compared to hearing/seeing the score during the consultation?”
- “Did you understand what the [practice nurse/healthcare assistant] meant?”
- “What did you think about the way in which the Health Professional communicated your score?”
  - *“What the Health Professional said/how they said it?”*
- “Were the risk results what you expected?”
  - *“How did this make you feel?”*
- “Is this how you remembered the conversation?”
  - *“If no, can you explain what you thought was different?”*
- “Did you find this information useful?”
  - *“What was useful/not useful about the information?”*

(If manipulation **was** used):

- “Do you remember being shown how your cardiovascular disease risk might change if you made some changes to your lifestyle?”
  - *If yes, what were the main messages you took away from this part of the Health Check?”*

**[Show video excerpt/s]**

- “Having watched this again, is there anything you now see but had not previously remembered?”
- “How did this information make you feel at the time?”
- “Did you understand the potential impact of [lowering blood pressure/cholesterol, stopping smoking etc] on your cardiovascular disease risk?”
  - “*Was there anything in particular that you did not understand?”*
  - *“What would have been helpful to you to help you understand this?”*
- “Did you find this information useful/not useful? Why?”
- “Were you happy with the level of information received?”
  - *Just right/too little/too much?*
- “Did you share information about your level of cardiovascular disease risk with a family member or friend?”
  - *“If you did, what did you tell them (if you don’t mind me asking)?”*
  - *“Did you feel confident in telling them what it meant?” “Why?”*

1. Follow up – Interventions & Recommendations

- “Has being told your risk for developing cardiovascular disease affected you in any way?”
  - *“If yes, how?”*
- “Can you remember what recommendations or suggestions were made by the Health Professional during your Health Check?”
  - *If yes, what were they?”*

**[Show video excerpt of all recommendations made by health professional – introduce each section]**

- “I know it’s only been a short while since your Health Check, but have you started to carry out some of the suggestions?”
  - *“If no…”*
    - *“Have you thought about it?”*
    - *“Are there reasons why you have chosen not to?”*
  - *“If yes…”*
    - *“What changes do you hope to make?”*
    - *“What changes have you started to make?”*
      - *“How is it going?”*
    - *“If you have made changes or have started to think about making changes, what spurred you on to make them?”*
    - *“Have you received any support from family or friends when trying to make the changes?”*

1. Alternative Presentation of Risk Information

Heart Age and Lifetime Risk

- “The [health professional] described your risk of a heart attack/stroke using a percentage. There are other ways this can be given to you. You can also be given your Heart Age [show example] or your lifetime risk [show example]. These are examples for us to show you and are not reflective of your own risk.”
  - “*Would this have increased your understanding of your CVD risk?”*
    - *“If no, why?”*
    - *“If yes, which one? Why?”*
  - *“Would this information have been useful/not useful? Why?”*

If manipulation **was not** used

- “If the [health professional] showed you how your level of risk would decrease if you [recommended changes; i.e., increased your physical activity, improved diet – **show example**]…”
  - *“Would this information have been useful/not useful?”*
  - *“Would this have impacted on your intention to make the changes suggested?”*

1. Conclusion

- “From your experience of the Health Check how could it be improved in the future?”
  - *Signposting/level of information/way risk was talked about?*
- “Finally, have you got anything you want to add or anything you think we would find interesting that we haven’t already talked about?”

“Thank you for your time in taking part in our study. Would you be interested in receiving a summary of our findings once it has been completed?” [If yes take contact details]
